# Learning Electronic Polarization in Molecular Systems: Vibrational Spectroscopy of Ethanol–Water Mixtures

**DOI:** 10.1021/acs.jcim.6c00491

**Published:** 2026-04-23

**Authors:** Oliver S. Cunningham, David M. Wilkins

**Affiliations:** Centre for Quantum Materials and Technologies, School of Mathematics and Physics, 1596 Queen’s University Belfast, Belfast, Northern Ireland BT7 1NN, U.K.

## Abstract

Mixtures of water and ethanol are an important solvent for biochemical reactions, as well as being a model system for hydrophobic hydration, since the ethanol molecule consists of polar and nonpolar regions. Experiments carried out over the last several decades have not yet reached agreement on what the addition of ethanol does to the structure of water, with some studies finding evidence for highly ordered cage-like structures and others finding no such evidence. We combine machine-learning force fields for the interatomic interactions in systems containing organic molecules with models that we develop for polarization, and show that we obtain good agreement with experimental infrared spectra, whose results are thought to imply the formation of cage structures. We show instead that in our simulations there is a decrease in the structure of water close to ethanol molecules and that the experimental observations can be explained by the change in structure further away from the ethanol.

## Graphical abstract

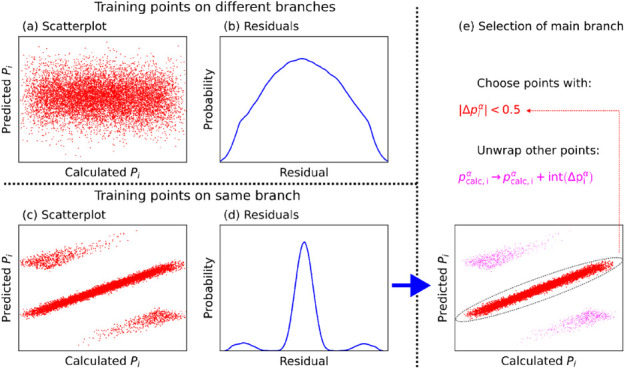

## Introduction

Mixtures of water and ethanol are important in biochemical processes such as the purification^1^ and helical structure-formation^2^ of proteins, as well as their dehydration.^3^ The differences in dielectric constants of pure water and pure ethanol mean that the dielectric constant of their mixture can be tuned over a wide range,^4^ making these mixtures a useful solvent. Solutions of ethanol in water are highly nonideal,^4,5^ with anomalous properties such as nonmonotonic dependence of the viscosity on mixture composition.^6,7^ These findings have been rationalized in terms of the hydrogen-bonding (H-bonding) structure in the mixture,^8–10^ particularly in terms of the existence (or otherwise) of cage-like clathrate structures in which the water molecules close to ethanol molecules are more structured than in pure water.^11,12^ However, the question of what kind of structure is formed in aqueous solutions of ethanol has not yet been definitively answered.^13^

Several studies have concluded that there is no evidence for clathrate structures in water–alcohol mixtures: neutron diffraction experiments,^14,15^ as well as classical^9^ and *ab initio* ^16^ molecular dynamics simulations found that dissolving ethanol in water did not affect the underlying H-bonding network. On the other hand, the Raman spectra of ethanol solutions showed enhanced hydrogen bonding compared to pure water,^17^ and water–ethanol clathrates are apparently observed in dielectric relaxation.^11^ Proton nuclear magnetic resonance (NMR) experiments found that above a critical concentration of ethanol, a peak attributed to an ethanol cluster, referred to as a “transient semi-clathrate structure”, appears.^18^ Since ethanol is a model system for hydrophobic hydration,^10,19,20^ it is clearly vital to understand the structure of its solutions.

Recent work has used infrared (IR) spectroscopy to conclude that adding ethanol to water leads to a change in the hydrogen-bond structure, with the formation of clathrate cages.^13^ This conclusion was supported by atomistic simulations of an ethanol molecule surrounded by a cluster of 30 water molecules, in which the latter formed a cyclic H-bonded structure. To make the link between the microscopic structure and dynamics of the water–ethanol system and the IR spectra would require long-time scale simulations of mixtures at varying concentrations, preferably using *ab initio* methods. However, these calculations are computationally very expensive.

The use of machine learning (ML) models for the interactions in chemical systems and interatomic forces has become a very popular method to avoid the need to use *ab initio* calculations, promising the accuracy of quantum mechanics using a fraction of the computational resources.^21–25^ To describe vibrational spectra also requires that the electric response of the system be known. In particular, the intensity of the IR spectrum at frequency ω is given in terms of the polarization ***P***,

1 $$I_{IR}\left(\omega \right)∼\omega^{2}\int \left\langle P\left(t\right)\cdot P\left(0\right)\right\rangle e^{i\omega t}dt$$

Methods for predicting the response of a system to an electric field have also become increasingly popular,^26–31^ including specialized methods for learning dipole moments^32–34^ and bulk polarizations.^29,35–37^ Either implicitly or explicitly, models for polarization must take into account the fact that when calculated using the modern theory of polarization,^38,39^ ***P*** is not a smooth function of the atomic positions: instead, the data is periodic.^40^

In ref 41, two methods were proposed to learn polarizations with the symmetry-adapted Gaussian process regression (SA-GPR)^28^ approach, in which kernel-based models are trained and the symmetry of the properties to be learned is built into the structure of the kernels. In the first method, the polarization data was preprocessed to render it a smooth function of atomic coordinates, by comparing it with a simple point-charge model, and in the second method the displacement of localized Wannier centers around each atom was learned, and the prediction used to build up the total polarization.^29^ Both of these methods gave data that could be learned straightforwardly, meaning that a practitioner in the field can use whichever method best suits the data they have available; however, it was not immediately apparent that these methods would work for systems containing molecules more complex than water.

In this paper, we further develop the two approaches of ref 41 to make them applicable to general molecular systems: in particular, the preprocessing approach no longer requires a point-charge model; instead, polarizations are processed using a two-step, data-driven method. We then use both approaches to build models for the polarization in water–ethanol mixtures and thus to predict how the IR spectrum changes as the concentration of ethanol changes. We find that ML models are able to capture the very subtle dependence of spectral features on ethanol concentration. By leveraging the ability of these models to predict molecular dipole moments we show that when the electronic structure of the water–ethanol solution is taken into account, the changes in vibrational spectra with enrichment or depletion of ethanol can be explained without invoking clathrate structures. Rather than water molecules in the first hydration shell of ethanol, it is the further-away molecules whose hydrogen-bonding is stronger, and it is these molecules that account for the concentration dependence of vibrational frequencies.

## Computational Details

### Molecular Dynamics Simulations

To generate a training set for our ML models, we ran several simulations using the GROMACS code,^42^ with systems containing 64 molecules: 17 different concentrations of ethanol in water were considered, from 0 to 64 of the molecules being ethanol and the remainder being water, and steps of 4 molecules between each concentration. For each concentration, 10 different simulations of 2 ns each were carried out after equilibration and 50 frames collected from each simulation, for a total of 8,500 frames (i.e., 500 frames for each concentration). The ethanol molecules were described by the OPLS/AA force field^43^ and the water molecules by the TIP4P/Ew model.^44^ Harmonic potentials were used to describe bonds in the molecules, rather than holding them rigid, to ensure that a sufficient range of configurations was explored.

The calculations were then used as input to the CP2K code,^45^ from which the energies and forces were computed, as well as the positions of Wannier centers and total polarization ***P***. These calculations were carried out using density functional theory (DFT), at the revPBE^46^ level, with Grimme’s D3 corrections.^47^ Full details on all calculations are given in the (Supporting Information SI), which provides input files, scripts and results for each step of the data generation process. We also show in the SI that our results are insensitive to the level of theory at which we calculate the polarization: the ***P*** calculated using revPBE-D3, which is a generalized-gradient approximation (GGA) functional, agrees extremely well with calculations at the revPBE0-D3 level of theory (i.e., a hybrid functional). Repeating our calculations with models trained at the hybrid level of theory, there is in all cases either no difference or a very minor quantitative difference from the GGA level.

For production molecular dynamics (MD) calculations, we used the MACE-OFF force field,^48^ which has been shown to give an excellent description of the properties of a wide range of organic molecules. These simulations were carried out in Python, with the mace package.^49^ Input files and scripts for these calculations are also provided in the SI.

All MD simulations were carried out without accounting for nuclear quantum effects (NQEs). While it is known that NQEs can have a significant effect on vibrational spectra, particularly for high-frequency vibrations,^50–52^ this effect tends to involve changes to the line positions and shapes, but not to make qualitative changes in the spectra such as changing the orders of peaks. This means that even though we do not expect that the frequencies of peaks in our IR spectra will necessarily be in the same position as in experiment, we will be able to assign and analyze them and to draw qualitative and semiquantitative conclusions from them.

### Learning Periodic Data

In the modern theory of polarization, the polarization ***P*** is defined modulo a “quantum of polarization” ***Q***,^38,39^ proportional to the unit cell of the system. In other words, the elements of the reduced polarization ***p*** = ***Q*** ^–1^ ***P*** lie between −1/2 and +1/2. For a reduced polarization component *p* ^α^ close to +1/2, a small change in the atomic configuration can lead to the polarization changing to −1/2, and vice versa. This large, discontinuous change in the polarization adds an extra layer of complexity to learning ***P***. The following subsections describe the two methods we used to overcome this problem, building on previous work.^41^

Given the periodicity of the polarization, the appropriate error function to gauge the quality of our ML models is the von Mises error function,^53^

2 $$\mathrm{vME}=1-\frac{1}{3N}\overset{N}{\underset{i=1}{\sum }}\overset{3}{\underset{\alpha =1}{\sum }}\cos\left[2\pi \left(p_{\mathrm{pred},i}^{\alpha }-p_{\mathrm{calc},i}^{\alpha }\right)\right]$$

where $p_{\mathrm{pred},i}^{\alpha }$ is the α^th^ Cartesian component of the predicted reduced polarization for the *i*th training point, $p_{\mathrm{calc},i}^{\alpha }$ is the analogue for the calculated polarization, and *N* is the number of data points.

In both of the approaches we will take to building polarization models, the quantity we wish to learn is a vector. To learn these properties we use symmetry-adapted Gaussian process regression (SA-GPR),^28,30^ which generalizes standard Gaussian process regression (GPR)^54^ to allow the modeling of data that transform as a tensor under rotations. We will use the λ-SOAP (smooth overlap of atomic positions) kernel of ref 28, which generalizes the scalar SOAP kernel.^55^

### Data-Driven Preprocessing of Polarizations

Because the physics of a periodic system is invariant under the change ***P***_pred,*i*_ → ***P***_pred,*i*_ + ***Q***_*i*_ ***n***, where ***n*** is any vector of integers,^38^ we can choose vectors ***n***_*i*_ that renders the polarization a smooth function of atomic positions. In ref 41, the vectors ***n***_*i*_ were chosen to minimize the difference between the calculated polarization and the prediction of a point-charge model. An alternative approach was also suggested that would allow ***n***_*i*_ to be found in a data-driven way, without the need for such a model; however, this data-driven preprocessing required a model for the derivatives ∂ ***P***_*i*_/∂*q*_*j*_ with respect to atomic positions, called the Born effective charges.

We describe in this section an alternative method that does not require derivatives, and so can be implemented without the need for any additional calculations. This also increases enormously the systems to which our existing methods are applicable. This improved approach is shown schematically in Figure 1 and described in detail below:

1. A small fraction (∼5%) of the training set is chosen at random and used to train an SA-GPR model for the polarization. This is used to predict the polarizations of the remainder of the set.
2. There are two possible outcomes:
  (a) If the subset used to train this model contains training points from different branches of the polarization, in which small changes in atomic positions do not necessarily correspond to small changes in ***P***, then the predictions of the model will be no better than random chance (see Figure 1a). This means that the histogram of the residuals will be unimodal (see Figure 1b). The process is restarted from step 1, with a new subset chosen.
  (b) If, instead, the training points in the subset all come from the same branch, the predictions of the model will look like Figure 1c: the model is a smooth function of positions, but the calculated polarization is not, meaning that branches can be identified in the scatterplot between the two. The histogram of residuals is multimodal (see Figure 1d). We continue to step 3.
3. For every point in the training set, the reduced residual vector Δ ***p***_*i*_ = | ***p***_pred,*i*_ – ***p***_calc,*i*_| is calculated. If the α^th^ component of this vector is less than 0.5, then the point is on the main branch and the polarization is not changed.
4. The reduced polarization of points not on the main branch are modified component-wise by $p_{\mathrm{calc},i}^{\alpha }\to p_{\mathrm{calc},i}^{\alpha }+\mathrm{int}\left(\Delta_{p_{i}}^{\alpha }\right)$ . This brings all points onto the main branch.

**Figure 1:**
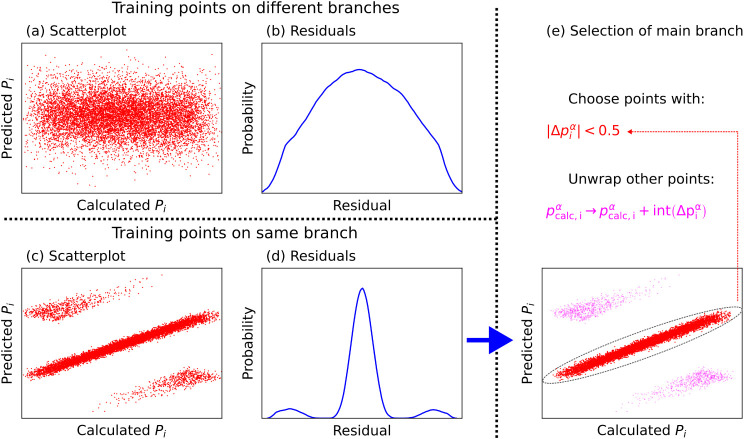
Schematic for unwrapping polarization data: (a) compares the predictions of a model trained on data points that come from different branches of the polarization with the calculated values; (b) shows the (unimodal) probability distribution of the residuals of this model; (c) compares the predictions of a model trained on data points from the same polarization branch with the calculated values; (d) shows the (multimodal) probability distribution of the residuals; (e) points that fall on the main branch are selected (red crosses) and used to build a model for the polarization. All points not on the main branch (magenta circles) are wrapped back onto the main branch by adding an integer multiple of the quantum of polarization.

The resulting data set contains reduced polarizations ***p***_*i*_ and polarizations ***P***_*i*_ = ***Q***_*i*_ ***p***_*i*_, that are smooth functions of positions and can be used to train an SA-GPR model. To decide whether the distribution of residuals was unimodal or multimodal we used the Hartigan dip test;^56^ a script for carrying out the preprocessing procedure is given in the SI. In a multimodal histogram of the residuals the different modes will be separated by integer multiples of the quantum of polarization, so there will be no problems with multiple overlapping modes. Because the models trained in step 1 above only need to be able to predict the relative branches of pairs of data points, they need not be very accurate models. This allows us to use a small fraction of the data set.

### Learning Wannier Center Displacements

An alternative approach to learning the polarization of a system is to focus instead on the positions of the localized Wannier centers, writing the total polarization as,^39^

3 $$P=e\overset{N_{a}}{\underset{i}{\sum }}Z_{i}R_{i}-e\overset{N_{w}}{\underset{j=1}{\sum }}2r_{j}^{W}$$

with *eZ*_*i*_ the charge of the *i*th nucleus (of which there are *N*_*a*_) and $r_{j}^{W}$ the position of the *j*th Wannier center (of which there are *N*_*w*_).

To make it possible to learn the Wannier-center representation with λ-SOAP kernels, which are built using features based on atom-centered local environments, the centers must be assigned to the atoms. We rewrite the polarization of eq 3 as,^41^

4 $$P=e\underset{i}{\sum }\left[\left(Z_{i}-N_{i}^{W}\right)R_{i}-\underset{j\in i}{\sum }2\delta_{ij}\right]$$

where $N_{i}^{W}$ is the number of Wannier centers assigned to the *i*th atom and $\delta_{ij}=r_{j}^{W}-R_{i}$ the displacement of the *j*th Wannier center from the *i*th atom.

In previous work on aqueous systems, Wannier centers were assigned only to oxygen atoms, with each being assigned 4 centers. The quantity,

5 $$\Delta_{i}=\underset{j\in i}{\sum }2\delta_{ij}$$

was then predicted using ML approaches.^29,41,57–59^

In water under ambient conditions, if each Wannier center is assigned to its nearest oxygen atom then each atom will be assigned 4 Wannier centers. However, For the ethanol–water mixture this approach is not guaranteed to give the same number of centers to each atom of the same type: in fact, we find that most, but not all, of the terminal carbon atoms in ethanol are assigned 4 Wannier centers and most of the central carbon atoms are assigned 2 Wannier centers. In around 5% of the molecules, each of these atoms are assigned 3 centers. Assigned in this way, the value of $N_{i}^{W}$ for each atom also varies.

Faced with the possibility of learning $N_{i}^{W}$ and Δ_*i*_ independently for each atomic center, we note that while a small change in a Δ_*i*_ value would lead to a small change in the total polarization, a Wannier center being spuriously transferred from one atom to another (by incorrectly predicting their $N_{i}^{W}$ values) would lead to a very large difference in the polarization: for two atoms separated by 1 Å, this would change the polarization by 9.6 D. It would also be necessary to ensure that the number of Wannier centers gives a total charge of zero, e.g., by using a version of the charge equilibration scheme.^60^

Since the Wannier centers are only assigned to atoms to make it possible to learn their positions, we are free to assign them as we wish. In this work, each Wannier center was first assigned to the nearest atom. Each atom was then labeled by the atoms within a small cutoff distance, and the most probable number of centers assigned to each atom found. In cases where the number of assigned Wannier centers was not equal to the most probable number, the center is found instead on a nearby atom; pairs of bonded atoms, one of which had a center fewer than the most probable number and one of which had a center more, were identified, and a center reassigned from the latter to the former atom. Along with the SA-GPR model for Wannier displacement Δ_*i*_, our overall model also comprises a list of the number of Wannier centers assigned to each type of atom label.

## Results and Discussion

### Machine Learning Models

We trained models for the polarization of ethanol–water mixtures. Figure 2a shows the learning curves for these models, with up to 8000 frames used to train the model and the remaining 500 points used to calculate the von Mises error. The polarization-unwrapping and Wannier displacement learning methods perform quite differently: while the two models give very similar errors for a small number of training points, the error for the unwrapping method continues to decrease steadily as training points are added to the model, whereas the Wannier displacement prediction quickly saturates. Although the error of the former method is around 10 times smaller than that of the latter when the full training set (8000 frames) is used, the scatterplots of Figure 2b and c show that both models give very good agreement between the calculated and the predicted data. We investigate in later sections how the differing performance of the two models in calculating the polarization translates to the infrared spectrum, which is derived from the polarization. It should be noted that the scatterplots compare the calculated and predicted values of cos­(2*π p*_*i*_), with *p*_*i*_ the elements of the reduced polarization; for circular data, it is these quantities that should be linearly correlated. We show in the Supporting Information that as well as giving excellent predictions for the independent testing set in Figure 2, our models trained on configurations obtained from force field simulations transfer extremely well to configurations from MACE-OFF simulations.

**Figure 2:**
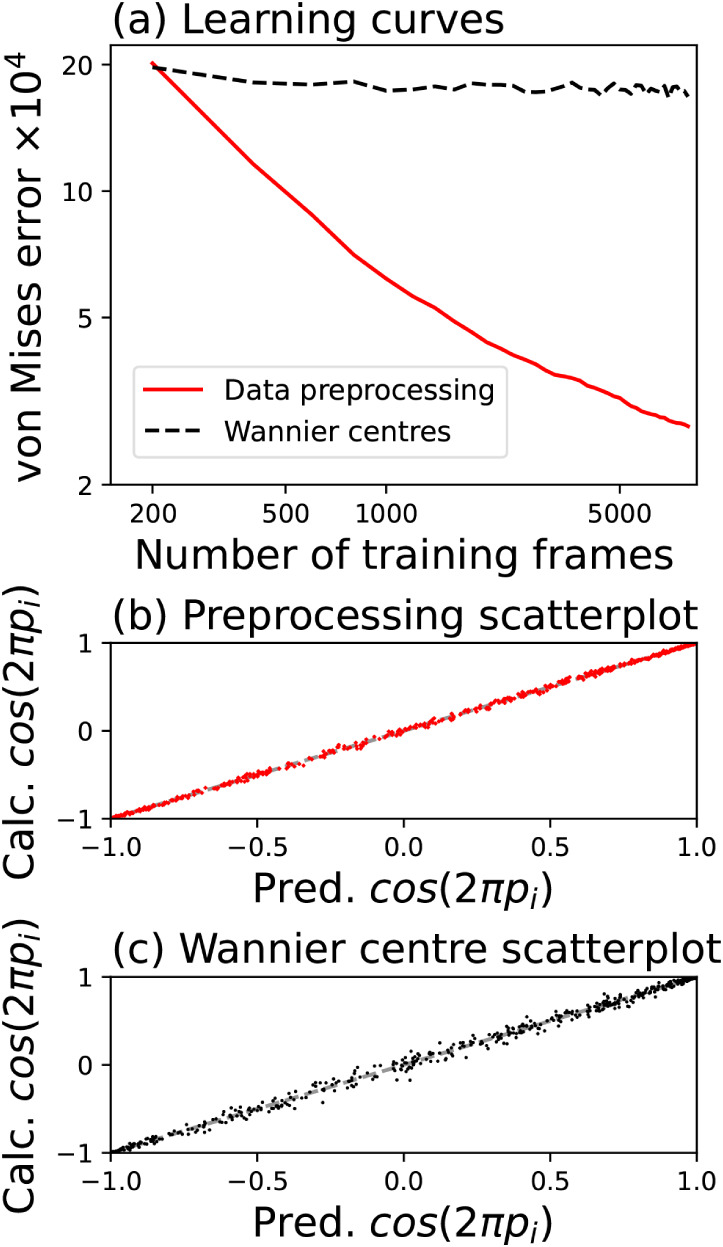
(a) Learning curves for the von Mises error (eq 2) of the polarization as modeled using either unwrapped data (solid red line) or Wannier center displacements defined in eq 5 (dashed black line); (b) Scatterplot for unwrapped data; (c) Scatterplot for polarizations from Wannier displacements. In the latter two cases, the scatterplots compare the predicted and calculated cos­(2*π p_i_*), where *p_i_* are elements of the reduced polarization.

Given the similar performance of the two models, rather than indicating that either of the models is substantially better than the other, Figure 2 reports on how the quality of the models changes as data is added. In ref 41, we found that for small training sets the Wannier center models outperform the preprocessed polarization models; this is also reflected in Figure 2a, and is likely to be a simple consequence of the fact that because there are between 192 and 576 atoms in each frame, depending on the concentration of ethanol, the effective number of training points for the Wannier center model is 2 orders of magnitude larger. Reference 41 also showed that for moderate numbers of training frames the models trained on preprocessed data perform better; we show in this work that for ethanol–water mixtures the trend continues at large numbers of training points. On the other hand, models for the Wannier displacements saturate quickly, with the addition of more training points adding little new information after a certain point. This result persists over a range of model hyperparameters, and is also seen for the error in the Wannier displacements **Δ**_*i*_ themselves, whose prediction error saturates early. We interpret this saturation as an indication that short-ranged, three-body features do not provide the optimal description of a system for learning Wannier displacements.

Our findings should not be taken to mean that it is *always* better to learn preprocessed data than to learn the positions of Wannier centers. Rather, the SA-GPR approach we have used with λ-SOAP features favors the preprocessing approach. We will discuss the relative performance of the two models for derived quantities in the following sections; for now, it is worth noting that predicting Wannier displacements has proven an excellent method to obtain infrared^29^ and sum-frequency generation^58^ spectra, and that a model for the positions of Wannier centers also gives an idea of the distribution of electrons in a system. Given that the approach in ref 29 gives a lower error for the polarization than in our work, albeit with several orders of magnitude more data, this suggests that a different choice of architecture or features may avoid the saturation we have seen.

### Infrared Spectroscopy

Figure 3 compares the experimental IR spectra of pure water and of a water–ethanol mixture with 50% mole fraction of ethanol^13^ with the spectra predicted using the preprocessed polarization model and the Wannier displacement model. We first note that for both concentrations, the two polarization models give spectra that agree very well on the frequencies of the peaks, but which disagree on their intensities. In particular, the relative height of the low-frequency (<1000 cm^–1^) compared to the higher-frequency peaks is much larger for the preprocessed polarization than for the Wannier center predictions. In the SI, we show that this trend persists over the concentration range we have studied.

**Figure 3:**
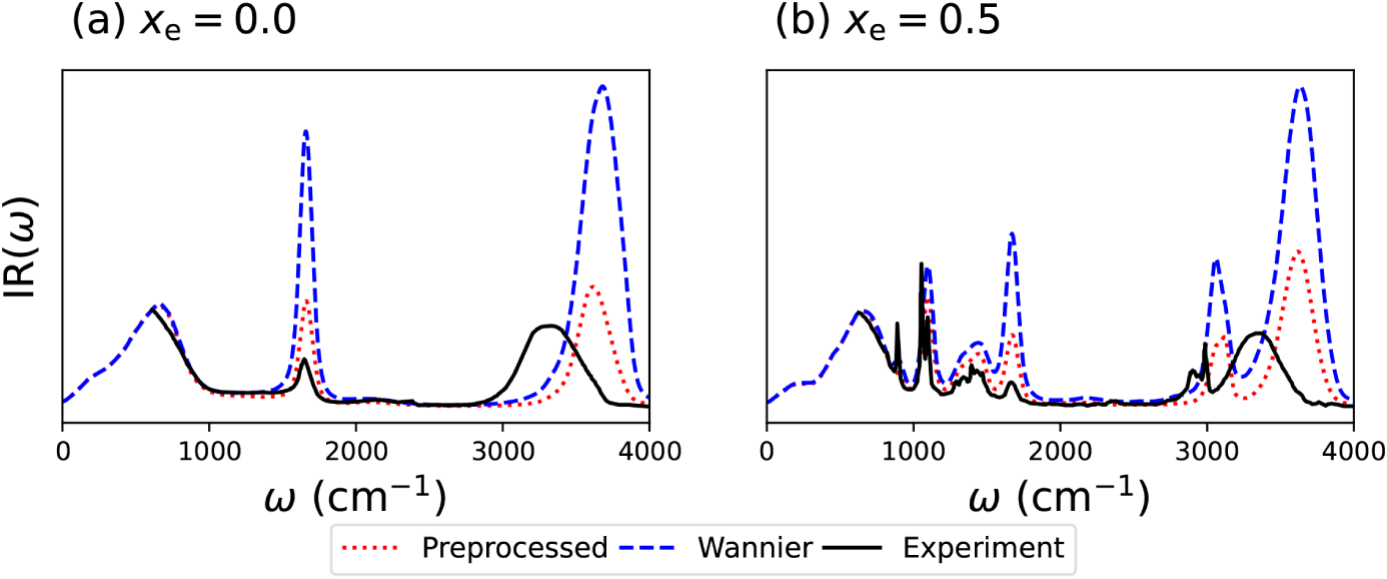
(a) Infrared spectrum of pure water; (b) Infrared spectrum of 50:50 water:ethanol mixture by number (80% volume fraction of ethanol). In all cases, red dotted lines show the results of a model trained using preprocessed polarizations, blue dashed lines the results of a model trained on Wannier center displacements, and solid black lines the experimental results, adapted with permission from ref 13. Copyright 2021 Elsevier.

To understand which of the two models is performing better, Figure 3 also shows the experimental IR intensities for the two mixtures.^13^ The first thing to note in this comparison is that while the positions of the low-frequency peaks are very well captured by our two models, the higher-frequency peaks (the stretching peaks involving H atoms) are blue-shifted compared to the experiments. This is unsurprising, as we have used purely classical molecular dynamics simulations. Taking into account the quantum nature of nuclei using path-integral simulations is expected to lead to better agreement with the experimental peak positions.^50,51^ Since our main interest is in understanding the extent to which the IR spectrum can be used to understand clathrate formation, a perfect agreement with experiment is not necessary. Nevertheless, it is heartening to note that the MACE-OFF potential gives an excellent description of the lower-frequency peak positions across the range of ethanol mole fractions.

Comparing the peak intensities of the preprocessed polarization and the Wannier displacement IR spectra with those of experiment, we see that the former model gives a much better description of the heights of high-frequency relative to low-frequency peaks than does the latter model. This indicates that, although both models give a very good performance for the polarization, the fact that the preprocessed polarization model has a lower error than the Wannier center model, as in Figure 2, does have a measurable effect on the spectrum, as resolving higher-frequency vibrations requires the (small) difference in polarization between two very similar configurations to be resolved.

Reference 13 used the frequency dependence of two vibrational modes to argue for the existence of clathrate structures: a peak at ∼1650 cm^–1^ in the experimental spectra (∼1660 cm^–1^ in computed spectra) corresponding to H–O–H bends in water molecules, and a peak at ∼3020 cm^–1^ in experiments (∼3060 cm^–1^ in computed spectra) corresponding to the C–H stretch at the β-carbon of ethanol. In experiments, the H–O–H bending peak shifts toward the blue end of the spectrum and decreases in magnitude as the concentration of ethanol increases, attributed to the disruption of the water hydrogen-bond network by ethanol molecules^8,13^ as the hydrogen-bonds weaken, the force constant for the H–O–H bend becomes smaller. The C–H stretch shifts toward the red end of the spectrum and increases in frequency with increasing concentration of ethanol; this is attributed to a shift of electron density from the methyl group toward the oxygen as the fraction of ethanol increases.^8^

Figure 4a shows the frequency of the H–O–H bend and Figure 4b the frequency of the β-C–H stretch as a function of the volume fraction *v*_e_ of ethanol. Since the computed peaks do not match perfectly with the experimentally measured peaks, we report the difference Δω between the peak position at a given concentration and the same position at either *v*_e_ = 0 (for the H–O–H bend) or *v*_e_ = 0.8 (for the C–H stretch). In both panels, the correct trend is predicted by both models: in Figure 4a the two polarization models are in excellent agreement with each other; while they predict a blue shift of only 11 cm^–1^ going from pure water to 80% ethanol by volume, less than the 17 cm^–1^ observed experimentally, the trend is qualitatively in very good agreement. As noted above, the disagreement between our simulated peak positions and those obtained from experiment are not surprising given that we have not accounted for NQEs. Given that Figure 4 shows the relative peak positions, we expect that the main effect of NQEs, if any, would be to change the gradient of the lines by a small amount, corresponding to a differing NQE for different concentrations. While this is an interesting avenue for future research, we note that these changes in gradient would not affect our ability to draw conclusions about the existence or otherwise of clathrates.

**Figure 4:**
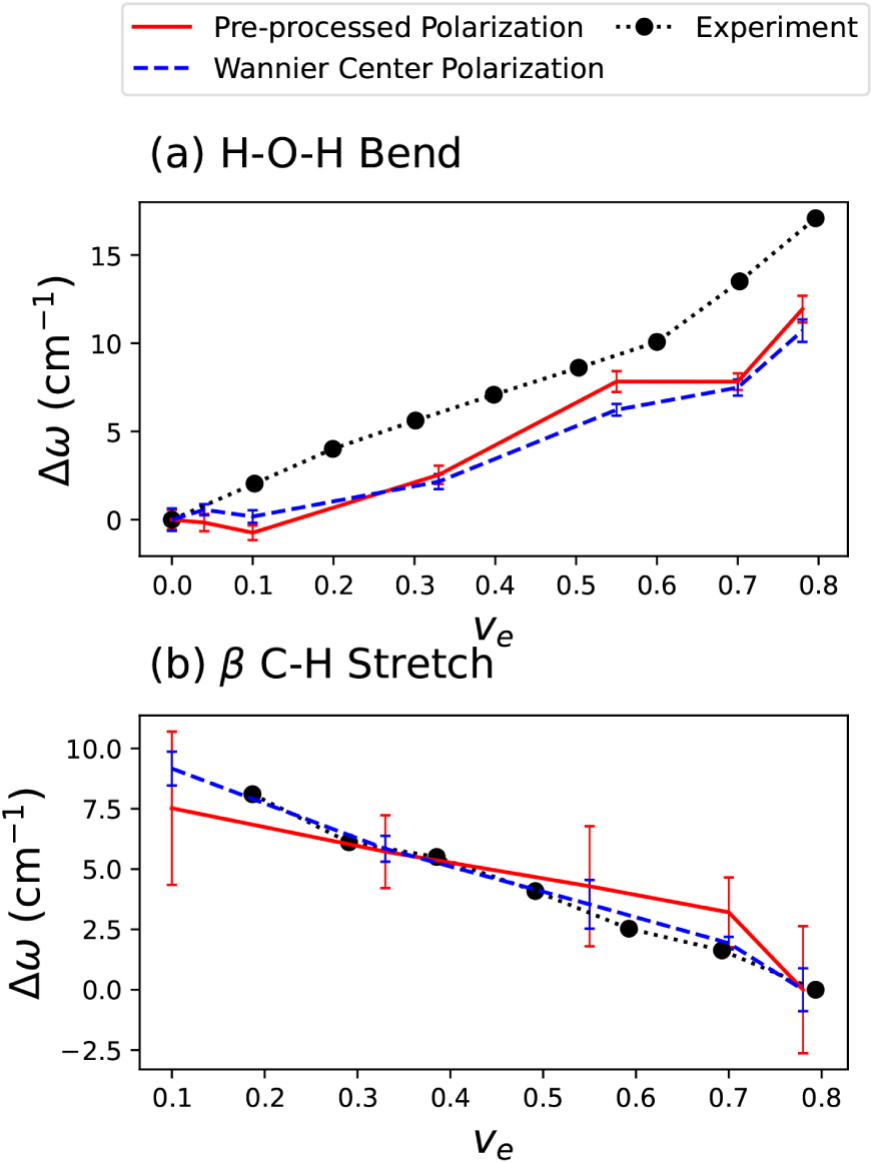
(a) Position of the H–O–H bending frequency in IR spectra as a function of ethanol volume fraction *v*_e_. (b) Position of the β-C–H stretching frequency as a function of *v*_e_. The peak position is taken relative to either its value at *v*_e_ = 0 (for panel (a)) or to its value at *v*_e_ = 0.8, the highest volume fraction for which there are results (for panel (b)). Red solid lines show the results of a model trained using preprocessed polarizations, blue dashed lines the results of a model trained on Wannier center displacements, and dotted black lines the experimental results, adapted with permission from ref 13. Copyright 2021 Elsevier.

For the C–H stretch, both of the polarization models predict overall a red shift, but while the Wannier center model agrees excellently with experiment, the preprocessed polarization model gives poorer agreement. This is an interesting counterpoint with the results of Figure 3, in which the Wannier center models compared less well with experiments than the preprocessed polarization models did. In fact, not only are the predictions of the peak positions larger for the preprocessed polarization model, but its error bars are much larger, indicating a less reliable model overall for this vibrational mode. However, despite this discrepancy, since both models give qualitatively good agreement with the experimental trend, we will be able to investigate both further to understand the extent to which clathrate formation is responsible for the experimental results.

### The Structure of Hydrated Ethanol

Because the two polarization models give good qualitative and semiquantitative agreement with the concentration-dependence of the key features in the IR spectrum, we can use these models with the MACE-OFF model to examine the claim that the experimental findings imply the formation of clathrate structures. Table 1 shows the average number *n*_HB_ of hydrogen-bonds formed by water molecules in ethanol–water mixtures; this number is also compared to the ideal number of H-bonds, $n_{\mathrm{HB}}^{\mathrm{id}}$ , which is the number that would be formed if there were a linear relationship between *n*_HB_ and the mole fraction *x*_e_ of ethanol. We note that the number of H-bonds formed is greater than the number that would be formed in ideal mixing, implying that additional structuring is taking place.

**Table 1:** Average Number of Hydrogen Bonds *n*_HB_ in Which a Water Molecule is Participating as a Function of the Mole Fraction *x*_e_ of Ethanol^a^

*x*_e_	*n*_HB_	Δ*n*_HB_
0.000	3.430(4)	0.00
0.016	3.431(4)	0.007(4)
0.032	3.425(4)	0.07(4)
0.125	3.421(6)	0.041(6)
0.250	3.37(1)	0.04(1)
0.375	3.31(1)	0.03(1)
0.500	3.23(1)	0.000

a $\Delta n_{\mathrm{HB}}=n_{\mathrm{HB}}-n_{\mathrm{HB}}^{\mathrm{id}}$ is the difference between the average number of hydrogen bonds and the ideal number (obtained by assuming a linear dependence on ethanol mole fraction). Numbers in brackets show the standard error in the final digit.

By itself, this finding does not imply the formation of clathrates: ref 19 used MD simulations to show that the number of hydrogen bonds is greater than that expected from ideal mixing in ethanol–water mixtures, but that the additional structure does not come from the water molecules nearest to the ethanol. Instead, it is important to resolve spatially where the additional structure is found. Figure 5 shows the local density of H-bonds in which a water molecule participates, as a function of their distance from the nearest carbon atom. The local density is calculated for three different mole fractions, particularly at *x*_e_ = 0.03, where the literature is in general agreement that increased structuring is seen.^61^

**Figure 5:**
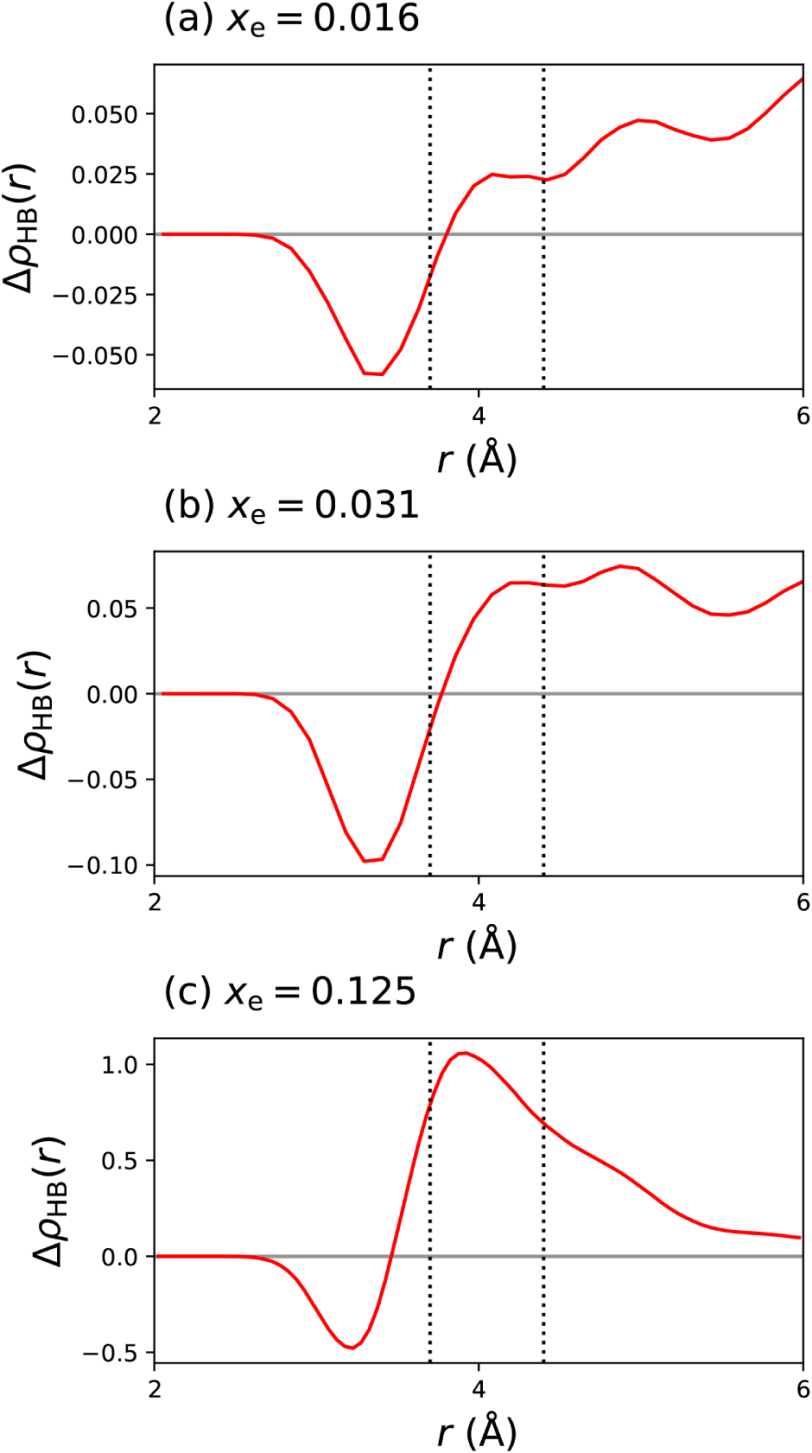
Excess number of hydrogen bonds per unit volume Δρ_HB_(*r*) for water molecules as a function of their distance from the nearest ethanol β-carbon, in an ethanol–water mixture with (a) mole fraction *x*_e_ = 0.03 of ethanol (2 ethanol:62 water); (b) *x*_e_ = 0.125 (8 ethanol:56 water); (c) *x*_e_ = 0.250 (16 ethanol:48 water). The excess number of H-bonds is the difference between the number of H-bonds in which the molecule participates and the ideal number, calculated by assuming a linear dependence of hydrogen bond number on *x*_e_. The first vertical line shows the position of the first maximum in the C–O radial distribution function and the second vertical line shows the position of the first minimum.

In agreement with ref 19, we see that rather than water molecules in the first hydration shell of ethanol forming more hydrogen bonds, they form *fewer*; the density of H-bonds remains below the ideal density until close to the edge of the first shell. The increase in structuring is from water molecules outside of the first shell of ethanol. As the ethanol concentration increases, the depletion of water molecules around the ethanol is greater because space is filled more with ethanol molecules, but the accretion in further coordination shells also increases. For Figure 5c the density of H-bonds already tails off by 6 Å, because the lower concentration of water means that there are fewer coordination shells of water.^19^

We also show the local density for higher concentrations (*x*_e_ = 0.125 and *x*_e_ = 0.250), which have a destructuring in the first shell and an increase in the number of hydrogen bonds in further shells, indicating that this effect persists over quite a high range of concentrations. At higher concentrations, it is not possible to identify coordination shells of water around ethanol beyond the first, due simply to the lower concentration of water.^19^ The highest distance at which we have calculated the excess density of H-bonds represents around half the simulation box lengths; to check that our results are not affected by finite-size effects, we have performed some simulations with 2 × 2 × 2 supercells. As shown in the Supporting Information, our results are robust to system size.

The models we have developed allow us to split the polarization into contributions from different parts of the system. The polarization can be rewritten ***P*** = ***P***_1_ + ***P***_2_, where ***P***_1_ is due to water molecules in the first shell of ethanol molecules and ***P***_2_ is due to water molecules that are not in the first shell of any ethanol molecules. For the Wannier center model ***P***_1_ and ***P***_2_ can be found by carrying out the sum in eq 4 either only over first-shell or over nonfirst-shell molecules. It is also possible to calculate ***P***_1_ and ***P***_2_ using the preprocessed polarization model: the predicted polarization for an atomistic frame is given as a sum of atom-centered polarizations (i.e., dipole moments),^41^ which can be partially summed either over atoms in first-shell water molecules or in nonfirst-shell molecules. The IR spectrum of eq 1 can then be written,

6 $$I_{IR}\left(\omega \right)=I_{1}\left(\omega \right)+I_{2}\left(\omega \right)+I_{c}\left(\omega \right)$$

where *I*_*i*_(ω) is the IR spectrum calculated using only the autocorrelation function of ***P***_*i*_(*t*) and *I*_c_(ω) the spectrum due to cross-correlations between the two quantities.

Table 2 shows the frequencies ω_*i*_ and intensities *I*_*i*_ of the H–O–H bending mode obtained from the spectrum *I*_*i*_(ω), for *i* = 1 (water molecules in the first shell of an ethanol molecule) and for *i* = 2 (water molecules not in the first shell of any water molecules), for the polarization predicted using Wannier center displacements. Most of the blue shift in frequency with increasing ethanol concentration is due to water molecules not in the first shell of ethanol molecules; over the concentration range shown the vibrational frequency increases by 17 cm^–1^, which is in fact the observed shift in the experimental spectrum due to *all* water molecules; there is a much smaller change in the frequency of water molecules in the first shell of ethanol.

**Table 2:** Frequency ω_i_ and Intensity *I_i_* of the H–O–H Bending Mode in the Water–Ethanol Infrared Spectrum Defined in eq 6 as a Function of the Mole Fraction *x*_e_ of Ethanol (*n*_e_ is the Number of the 64 Molecules That are Ethanols), with *i* = 1 Corresponding to Water Molecules in the First Hydration Shell of Ethanol and *i* = 2 Corresponding to Molecules Outside of the First Shell^a^

		Wannier center				Preprocessed polarization			
*x*_e_	*n*_e_	ω_1_ (cm^–1^)	*I*_1_	ω_2_ (cm^–1^)	*I*_2_	ω_1_ (cm^–1^)	*I*_1_	ω_2_ (cm^–1^)	*I*_2_
0.02	1	1665	1.00	1660	1.00	1667	1.00	1662	1.00
0.03	2	1665	1.78	1660	0.87	1667	1.65	1662	0.82
0.13	8	1665	4.24	1662	0.36	1667	2.63	1664	0.27
0.25	16	1666	4.76	1668	0.13	1669	2.01	1671	0.08
0.38	40	1667	4.29	1672	0.07	1671	1.29	1678	0.03
0.50	32	1670	3.61	1677	0.04	1674	0.84	1682	0.02

a In all cases, *I_i_* is given relative to the intensity for the lowest reported mole fraction. “Wannier centre” refers to spectra calculated using polarizations from the predicted Wannier displacement and “Preprocessed polarization” refers to spectra calculated using partially resummed atomic dipole moments.

Our findings reinforce those shown in Figure 5 and support those of ref 19, in which similar conclusions were drawn using classical force fields: while there is a measurable increase in H-bonding strength as ethanol is added to water, this increase comes from water molecules that are not in direct contact with ethanol, but rather are outside of its first hydration shell. Another interesting point to note is that the intensity of the peak in the *I*_2_(ω) spectrum decreases with increasing ethanol mole fraction: as the number of ethanol molecules increases there are simply fewer molecules that are not in the hydration shell of any ethanol. The intensity of the ω_1_ peak, on the other hand, increases with mole fraction for low *x*_e_: as more ethanol molecules are added, more of the water molecules are close to an ethanol. For large *x*_e_ the intensity begins to decrease again, reflecting the decreasing number of waters.

While we found in ref 41 that in pure water the molecular dipole moments calculated by partially resumming either for Wannier centers or for preprocessed polarizations were in good agreement with each other, the splitting of the preprocessed ***P*** into atomic contributions is purely data-driven and there is no reason that it should be in agreement with the Wannier-center prediction. The two methods, however, are in excellent qualitative agreement: the blue shift seen experimentally is mainly due to water molecules outside of the first shell of any ethanol molecules, while the peak frequency due to first-shell molecules changes only by a small amount. The final word on electronic structure and on charge distribution is, of course, given by the Wannier center model, which has been trained specifically to partition the charge. However, the data-driven partitioning of the preprocessed polarization model can point us in the right direction and provide a useful hypothesis to be tested further using Wannier centers. It may also be possible to distinguish the contribution to the IR spectrum from inner-shell and outer-shell water molecules using the method of multivariate curve resolution (MCR);^62^ if so, this could provide an independent verification of our findings.

We can also investigate further the β-C–H stretching mode in ethanol and its concentration dependence: while the red-shift in stretching frequency with increasing ethanol concentration has been ascribed to clathrate formation,^13^ there is not a clear relation between the presence of clathrates and this shift. In the SI, we show that while the coordination number of oxygen atoms around a C–H methyl bond decreases with increasing ethanol content, the experimental dielectric constant decreases at a faster rate, meaning that there is a stronger electrostatic force pulling H atoms away from the β carbon, and a concomitant weakening of C–H bonds. This leads to a decrease in the stretching frequency. While this is in accord with the explanation given in ref 13, it does not require the existence of clathrate structures.

While the dielectric constant *ε*_r_ is an extremely difficult quantity to converge from atomistic simulations^63^ and the relatively short trajectories that we used in this work did not allow us to compute *ε*_r_ directly, we can nonetheless connect our results to dielectric trends. For example, Figure 4b shows that the preprocessed polarization model performs less well than the Wannier center polarization model in capturing the behavior of the C–H stretching peak. We have seen that this behavior depends sensitively on the dielectric constant of the solution, raising the possibility that the disagreement for the preprocessed model is due to an underlying failure in describing the dielectric constant. This will be the subject of future research.

A natural question to ask is whether the findings in this work are transferable beyond mixtures of water and ethanol to dilute solutions of other small, hydrophobic molecules in water. This is supported by experimental results: neutron diffraction experiments from dilute solutions of methanol show that the water in the first shell of methanol does not change its structure, while that in the second shell is slightly more structured,^64^ and the same is seen in solutions of tertiary butanol.^65^ Classical MD simulations of water around hydrophobic ion clusters also show evidence that the water molecules in the first shell of these neutral clusters form fewer H-bonds and that those in the second shell and further out form more H-bonds than in bulk water.^66^ This suggests that indeed our results have wide applicability, and that it would be interesting to study the IR spectra of systems with other small hydrophobic solutes, both experimentally and computationally, at the *ab initio* level of theory.

## Conclusions

In this paper, we have developed two symmetry-adapted machine-learning methods for learning polarization in condensed-phase systems: one in which the polarization training data is preprocessed so that it is a continuous function of positions, and one in which the average displacement of Wannier centers from atoms is predicted and used to find the total polarization. While the performance of the preprocessing method shows a better improvement with increasing training set size than that of the Wannier center method, both give excellent results for the total polarization. Applying both of these methods to mixtures of water and ethanol at varying mole fractions, we have shown that they perform quite differently in predicting the infrared spectrum. The Wannier center model has a higher error than the preprocessed polarization model, which appears at first sight to point to a poorer performance. However, the polarization errors of both models are extremely good, and the fact that the Wannier model gives the best description of experiment overall shows *post hoc* that it is a useful approach.

Combining our polarization models with the results of molecular dynamics simulations, we were able to investigate the claim that the dependence of features in the infrared spectrum on ethanol concentration could only be explained by the existence of clathrate structures, in which water molecules in the first hydration shell of an ethanol molecule participate in more hydrogen bonds than they would in pure water. We found instead that these first-shell molecules take part in *fewer* H-bonds, and that the concentration dependence of the spectral features should be ascribed to water molecules further away from ethanol.

In principle our preprocessing method is generally applicable, as it only requires a small number of configurations whose polarizations fall on the same branch; these can be used to build a model that identifies whether other configurations are on this branch. However, this initial step may prove difficult: if the system can switch between many possible branches with small energy barriers between them, such as for a system with free charges, then it may be difficult to find many configurations on one branch. Future work will focus on better understanding the possible limits of our methods, as well as applying them to higher-order responses.

## Supplementary Material



## Data Availability

Data and Software Availability Statement: The data used to train models, as well as details of quantum-mechanical calculations, the trained models, and instructions for applying them, can be found in the Supporting Information.

## Notes

Description of online repository, further details on model building, validation and transferability, as well as finite-size effects and the effect of varying the DFT functional (PDF)
