# Supplementary material for: Learning Electronic Polarization in Molecular Systems: Vibrational Spectroscopy of Ethanol–Water Mixtures
Source: J Chem Inf Model. 2026 Apr 23;66(9):5179–88. doi: 10.1021/acs.jcim.6c00491 (PMC13169363; doi:10.1021/acs.jcim.6c00491)
Supplement: Supplementary file 1 [file ci6c00491_si_001.pdf]

Supplementary Information

Learning Electronic Polarization in Molecular Systems: Vibrational Spectroscopy of Ethanol-Water Mixtures

Oliver S. Cunningham[1] and David M. Wilkins[1, a)]
Centre for Quantum Materials and Technologies, School of Mathematics and Physics, Queen's University Belfast, Belfast BT7 1NN, Northern Ireland, United Kingdom

CONTENTS

I. Calculation Details and Scripts 2
II. Comparison of Polarization Models for IR Spectra 2
III. C–H Stretching Frequency 2
IV. Model Transferability 2
V. Comparison of GGA and Hybrid Models for Polarization 4
VI. Finite-Size Effects 4
Bibliography 4

a) Electronic mail: d.wilkins@qub.ac.uk

I. CALCULATION DETAILS AND SCRIPTS

Input files for the calculations carried out in this work can be found in the `molecular_polarization_2026` subfolder of the `https://github.com/dilkins/polarization-learning` repository, including a script for testing multimodality in the pre-processed polarization method.

II. COMPARISON OF POLARIZATION MODELS FOR IR SPECTRA

Fig. S1 shows the predicted IR spectra for water-ethanol mixtures at several mole fractions x_{e} of ethanol. As in the main text, the ratio of high-frequency to low-frequency peaks is much higher for the spectrum predicted using the Wannier centre model than for the spectrum predicted using the pre-processed polarization model.

III. C–H STRETCHING FREQUENCY

To understand why increasing the ethanol mole fraction weakens the C–H bonds (and thus decreases the C–H stretching frequency), we model the effective local charge on C–H hydrogen atoms, due to surrounding oxygen atoms, as,

$$q(x_{\mathrm{e}}) \propto \frac{q_O N_{\mathrm{O}}(x_{\mathrm{e}})}{\varepsilon_{\mathrm{r}}(x_{\mathrm{e}})}, \tag{1}$$

where q_O is the effective charge of an oxygen atom, $N_{\mathrm{O}}(x_{\mathrm{e}})$ is the number of oxygen atoms surrounding the H atom, and $\varepsilon_{\mathrm{r}}(x_{\mathrm{e}})$ is the dielectric constant, where the latter two quantities are functions of the ethanol mole fraction. In other words, the charge exerted on a C–H hydrogen atom is proportional to the number of surrounding oxygen atoms and inversely proportional to the dielectric constant.

Table SI shows these quantities as a function of ethanol mole fraction, where $N_{\mathrm{O}} = \int_0^R 4\pi r^2 g_{\mathrm{HO}}(r)\,\mathrm{d}r$, with $R = 3$ Å and $g_{\mathrm{HO}}(r)$ the radial distribution function for oxygen atoms surrounding C–H hydrogen atoms. ε_{r} is taken from the experimental data of Ref.[1]. While the number of oxygen atoms surrounding an ethanol hydrogen atom decreases as the ethanol concentration decreases, a natural outcome of the decreasing concentration of water, the dielectric constant also decreases, and this occurs at a faster rate. This means that overall, the effective charge experienced by a C–H hydrogen atom due to the surrounding oxygen atoms *increases*, the hydrogen is pulled away from the carbon and the C–H bond is weakened, meaning that the frequency of the stretching vibration decreases.

x_{e}	N_{O}	ε_{r}	$10^3 \times N_{\mathrm{O}}/\varepsilon_{\mathrm{r}}$
0.016	0.418	75.2	5.56
0.031	0.415	73.3	5.66
0.125	0.410	62.1	6.61
0.250	0.371	51.0	7.28
0.375	0.340	43.0	7.90
0.500	0.307	37.0	8.30

TABLE SI: Average number N_{O} of oxygen atoms in the first shell of a C–H hydrogen atom, dielectric constant ε_{r}, and the quotient of the two, as a function of the mole fraction x_{e} of ethanol.

IV. MODEL TRANSFERABILITY

To validate the transferability of our polarization models, trained on configurations obtained from classical forcefield simulations for the sake of expediency, to configurations from MACE-OFF simulations, we took 7,500 frames at random from our MACE-OFF calculations. For each of these frames, we calculated the polarization using CP2K and predicted the same quantity using both of our models. Fig. S2 compares the predictions of the model with the calculated values, showing that both of our models give excellent predictions of the polarization: the preprocessed polarization model has an RMSE of 6% the deviation in the training set and the Wannier centre model has an RMSE of 9% of the deviation.

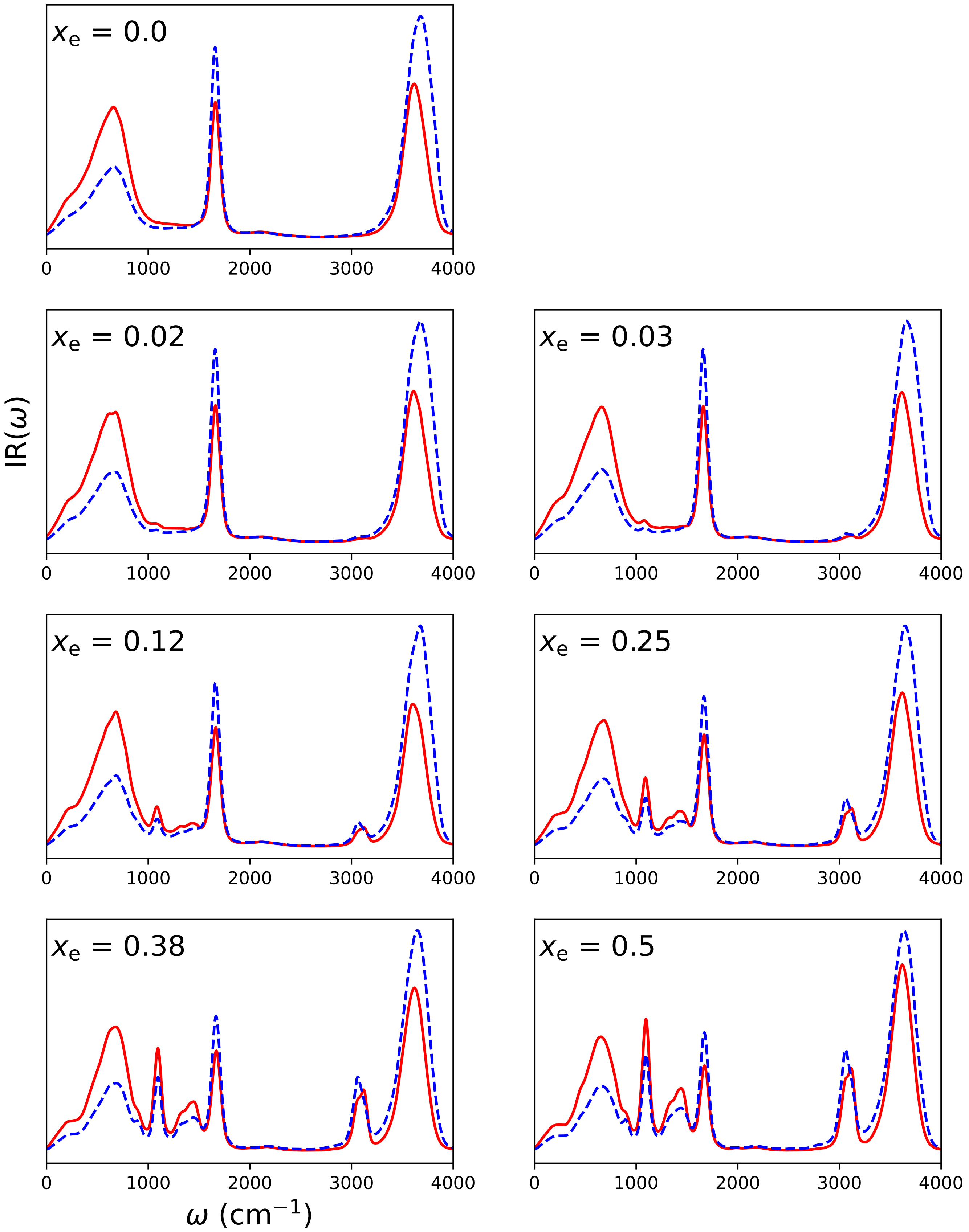

FIG. S1: Infrared spectra of mixtures of water and ethanol with varying mole fractions x_e of ethanol. Red solid lines show the results of a model trained using pre-processed polarizations, and blue dashed lines show the results of a model trained on Wannier centre displacements.

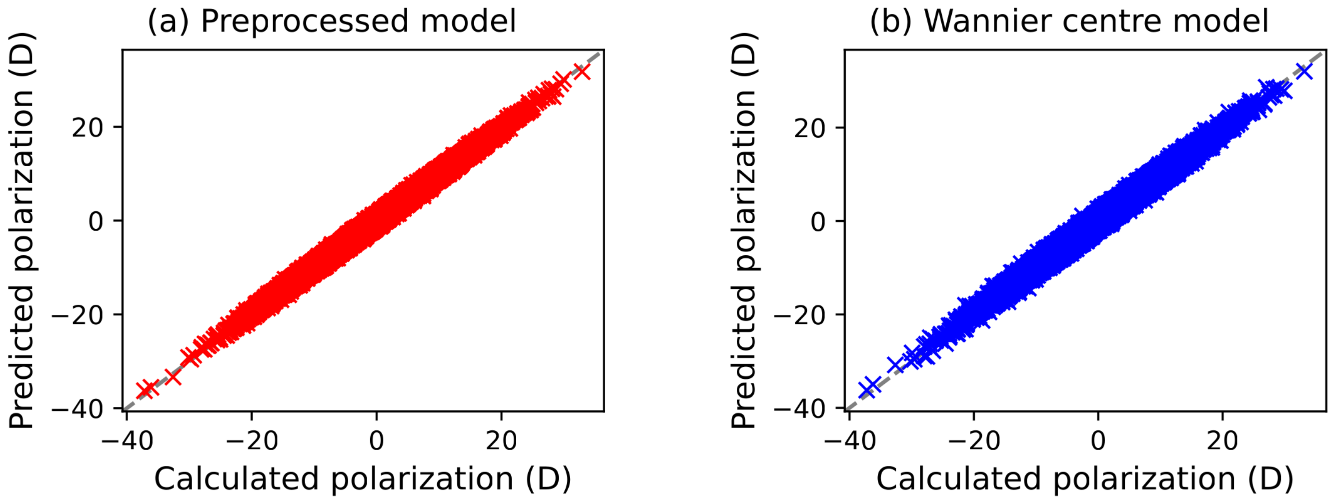

FIG. S2: (a) Scatterplot of predicted against calculated polarization using the preprocessed polarization model; (b) Scatterplot of predicted against calculated polarization using the Wannier centre model. In all cases, predictions and calculations are carried out on 7,500 frames taken from MACE-OFF calculations.

V. COMPARISON OF GGA AND HYBRID MODELS FOR POLARIZATION

To test the effect on our results of the level of theory we use for our polarization models, we have trained two models, one at the GGA level (revPBE-D3) and one at the hybrid level (revPBE0-D3) using the pre-processed polarization. Since we were limited in the number of hybrid calculations we could carry out, and to avoid the confounding effects of comparing models trained on two different sizes of system, we took 1,000 frames and trained models at both levels of theory. Fig. S3(a) compares the calculated polarizations for these 1,000 frames at both levels of theory: there is near-perfect agreement between the two. Fig. S3(c-d) compares the *predicted* IR spectra at the revPBE-D3 and revPBE0-D3 levels of theory for three mole fractions of ethanol: while the agreement between the spectra is not perfect, it remains excellent.

The positions of the H–O–H bend and C–H asymmetric stretch peaks are more subtle: even a small change in the spectra between the two levels of theory could make a relatively large change in the peak positions. Fig. S3(e-f) shows that predicted position of the H–O–H bend agrees extremely well between the two levels of theory, while the asymmetric C–H stretch does change a little at low volume fractions of ethanol; however, this small quantitative change does not make a qualitative difference to our conclusions.

VI. FINITE-SIZE EFFECTS

To test whether our results depend significantly on system size we repeated some of our calculations with a $2 \times 2 \times 2$ supercell. While the MACE-OFF model makes these calculations feasible, they are still computationally quite expensive with the resources available and we were not able to converge them to the same level as the results in the main text. Fig. compares the excess density of H-bonds made by water molecules surrounding an ethanol molecule for two concentrations shown in Fig. 5 of the main text, using the original simulation box and the doubled box. We see that while the quantitative agreement is not perfect, we draw the same conclusions from the simulations with a larger box.

BIBLIOGRAPHY

[1] F. Franks and D. J. G. Ives, Q. Rev. Chem. Soc. **20**, 1 (1966).

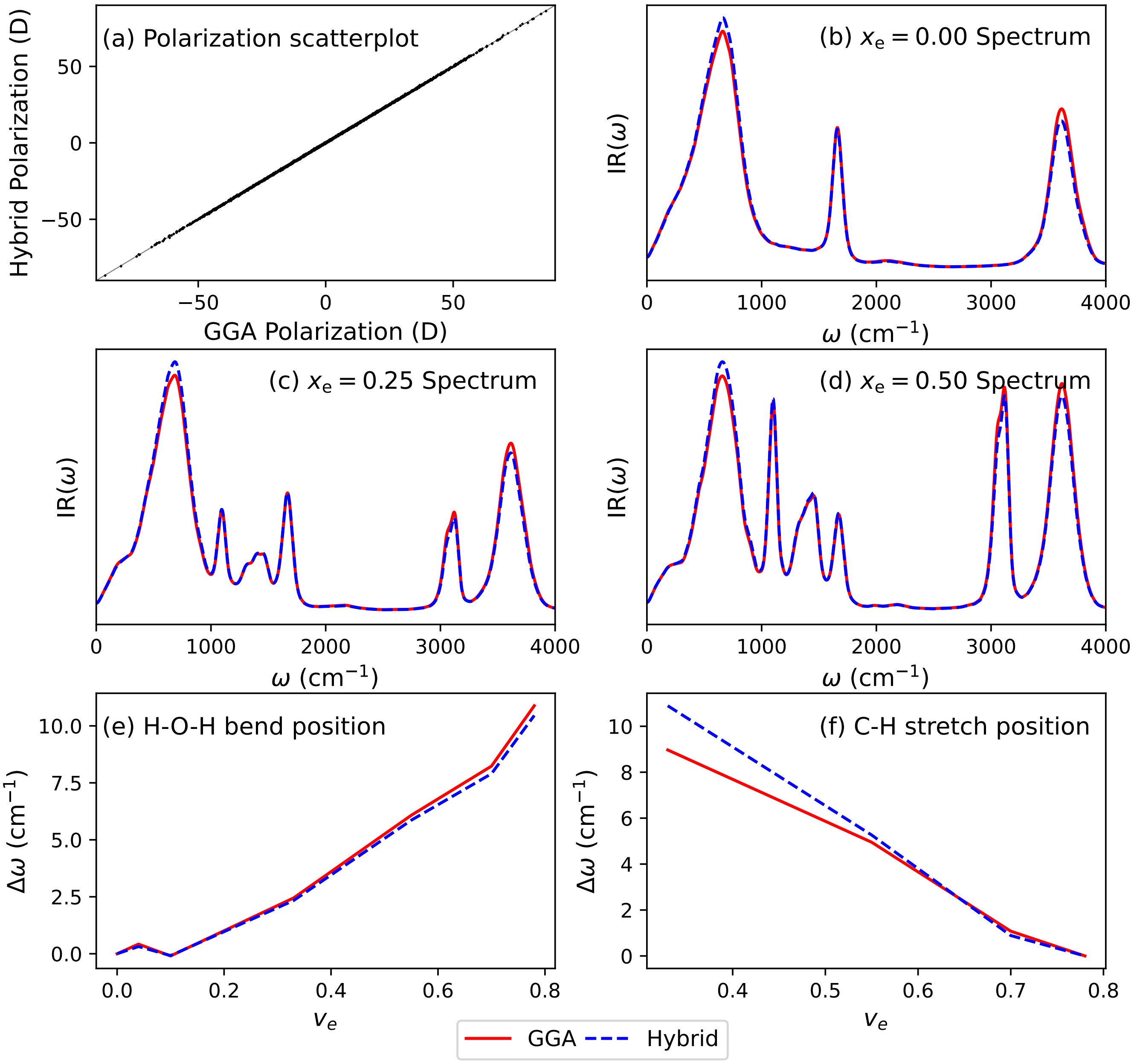

FIG. S3: (a) Comparison of polarizations calculated for 1,000 frames using the GGA functional revPBE with D3 corrections and the hybrid functional revPBE0 with D3 corrections; (b-d) Infrared spectra predicted using models built with pre-processed polarizations for revPBE-D3 (solid red lines) and revPBE0-D3 (dashed blue lines); (e-f) Positions of two vibrational peaks that we consider in the main text, from the predicted spectra.

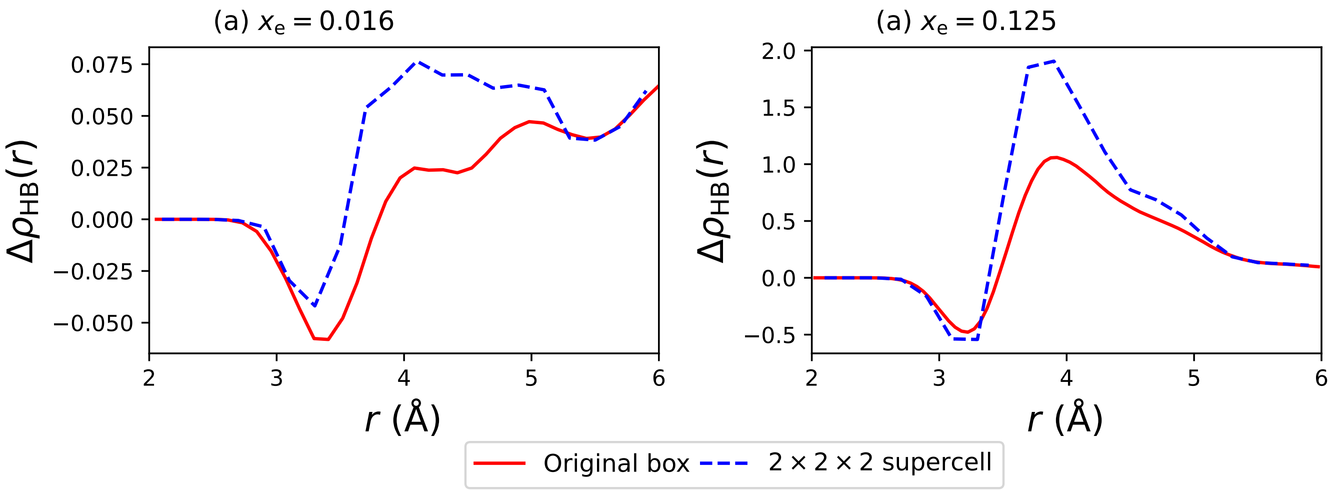

FIG. S4: Comparison of excess H-bond density mdae by water molecules at a distance r from an ethanol molecule for (a) $x_e = 0.016$ and (b) $x_e = 0.125$. Solid red lines show the results from Fig. 5 in the main text, and dashed blue lines show the results obtained for a $2 \times 2 \times 2$ supercell.
